# Protecting Data Privacy in the Age of AI-Enabled Ophthalmology

**DOI:** 10.1167/tvst.9.2.36

**Published:** 2020-07-06

**Authors:** Elysse Tom, Pearse A. Keane, Marian Blazes, Louis R. Pasquale, Michael F. Chiang, Aaron Y. Lee, Cecilia S. Lee, and AAO Artificial Intelligence Task Force

**Affiliations:** 1 Department of Ophthalmology, University of Washington, Seattle, WA, USA; 2 Medical Retina Service, Moorfields Eye Hospital NHS Foundation Trust, London, UK; 3 Institute of Ophthalmology, University College London, London, UK; 4 Eye and Vision Research Institute, Icahn School of Medicine at Mount Sinai, New York, NY, USA; 5 Departments of Ophthalmology and Medical Informatics & Clinical Epidemiology, Casey Eye Institute, Oregon Health & Science University, Portland, OR, USA

**Keywords:** Machine Learning, Artificial Intelligence, Privacy, Biomedical Ethics

## Introduction

Digital data privacy is a rapidly evolving concept in health care. As electronic records have replaced paper charts, and with the rise of “Big Data” and artificial intelligence (AI), this issue has become increasingly important. Big Data has been defined by the three V's: volume (large amounts of data), variety (data heterogeneity), and velocity (speed of access and analysis).^1^^,^^2^ Analyses of these large datasets have allowed for more powerful assessments of healthcare quality and efficiency with the goal of improving patient care.^3^ AI is a branch of applied computer science that uses computer algorithms to perform cognitive tasks that approximate human intelligence, such as clinical decision making.^4^ More specifically, deep learning, a subset of machine learning within the field of AI, has been particularly successful in training powerful algorithms for the classification of medical images and other high-dimensional data.^5^^–^^9^ Taken together, these approaches may offer many benefits for patients, including automated screening and triage of disease and treatment optimization. For example, AI-enabled screening of diseases such as diabetic retinopathy, retinopathy of prematurity, and glaucoma could improve early detection and treatment.^5^^,^^10^^,^^11^ Furthermore, AI has been used for future disease predictions, in areas ranging from acute kidney injury to age-related macular degeneration and diabetic retinopathy; in the future, such approaches could lead to better preventative strategies.^12^^–^^15^ The combination of Big Data and AI also offers many potential benefits for healthcare systems, including increased productivity with decreased costs, as well as reductions in medical error. New data privacy problems have arisen with the use of this technology, however, leading to concerns about the balance between innovation and privacy and the need for better data protection methods that can evolve along with Big Data and AI.

## Ethical Considerations

In the United States, the Belmont Report is the most widely recognized ethical framework for health care and the life sciences, and it serves as an essential reference for institutional review boards.^16^ The Belmont Report highlights three fundamental principles: respect for persons, beneficence, and justice. Two additional bioethical principles—non-maleficence (often translated as “first, do no harm”) and respect for autonomy—are also considered central to biomedical ethics and AI development.^17^^,^^18^ The Belmont Report is not directly applicable to secondary use of de-identified clinical data; in the report, when identifying information has been removed, the use of that data is no longer considered human subjects research. Nonetheless, its core principles are instructive. In particular, with regard to beneficence at a population level, some believe it is unethical to refrain from using clinical data to develop tools that have the potential to benefit others. In contrast, when considering both non-maleficence and respect for autonomy, others weigh the balance of both risks and benefits of such applications for an individual where said individual does not derive benefit.^19^ In the United Kingdom, this is recognized in the constitution of its National Health Service (NHS) which pledges “to anonymize the information collected during the course of your treatment and use it to support research and improve care for others.”^20^ However, with the use of increasingly large clinical datasets, maintaining data privacy and confidentiality—and thus respect for persons—is a challenge.

## Data Protection and Privacy

One of the main limitations of machine learning and deep learning approaches is their requirement for large datasets for development and testing—datasets that are typically an order of magnitude or even greater than those collected in most prospective clinical trials. Compared to other medical specialties (e.g., obstetrics), ophthalmology has benefited from the widespread availability of large, well-curated imaging datasets and thus is often seen as being at the forefront of AI-enabled health care.^21^ Although the availability of anonymized datasets has been a boon for technological advancement, it also represents a significant risk. The principle of beneficence requires that healthcare professionals “do no harm”; yet, breaches of patient privacy can cause major harms and can also have unintended consequences. These could potentially impact one's employment or insurance coverage^2^ and may even allow computer hackers to obtain Social Security numbers and personal financial information.^22^

Removal of all potentially identifiable information from large datasets can be a daunting task. In fact, it is now clear that, even with the most rigorous efforts, there will always remain at least a theoretical risk of re-identification.^23^ This is not an issue unique to ophthalmology, as it is now conceivable to apply facial recognition software to three-dimensional reconstructions of computed tomography of the head. In addition, features from the periocular region have been used to identify the age of patients using machine learning algorithms.^24^ Gender, age, and cardiovascular risk factors have been identified from fundus photographs.^13^ Even for datasets not involving medical images, and even without the use of advanced or future technologies, it may be possible to identify individuals by linkage with other datasets. This is particularly the case as patient information generally accumulates over time.^25^

## Data Sharing

Another problem related to privacy and AI is managing the exchange of data in an ethically acceptable way. AI typically requires specialized technical expertise and powerful computer resources. In the case of a rare disease, for example, consolidation of data from multiple institutions would be required. As a result, datasets must be shared outside of the institution in which they were generated. If executed poorly, such data sharing may increase the risk of data breaches.

Data sharing that involves major multinational corporations in the pharmaceutical and technology sectors is also of great concern. Monetization of clinical data is a trending topic lately, as evidenced by the oft-repeated phrase “data is the new oil.”^26^ These increasing relationships between healthcare companies and academic research data can heighten the risk of malicious privacy violations. Although detailed discussion of this issue is outside the scope of this article, the use of exclusive contracts or licenses that prohibit sharing of routinely collected clinical data is another cause for concern. Thus, exclusive arrangements that restrict or preclude the widest possible patient benefit for clinical data could undermine the Belmont principle of justice.

## Models for Consent

In many countries, research ethics authorities do not require individual consent for retrospective research on de-identified datasets. This is generally well accepted in ophthalmology, and the majority of ophthalmic clinical research including the AAO IRIS (Intelligent Research in Sight) Registry takes place using this model.^27^ However, this practice is sometimes questioned in the context of machine learning, where the clinical data themselves are used to develop algorithms. Many patients are supportive of the use of their data to improve health care and research but feel that they should be asked to give permission first.^28^ At first glance, this would appear to be the most ethically sound approach—it is certainly an appropriate model for interventional research studies such as clinical trials. However, this approach is cumbersome or not feasible for large, historical datasets of routinely collected data. There are also challenges in prospectively deploying such a consent model, particularly when seeking permissions from patients for unforeseen future uses of their de-identified data. It would not be true informed consent if patients are asked to sign up to extensive terms and conditions before each episode of care or to agree to future uses of their data about which they have not yet been “informed.”

The use of opt-in models has also been proposed as a lighter touch approach; however, this means that only the most engaged patients, who actively take steps to get involved, will be included. Of course, the act of de-identifying itself may require consent from the covered entity. There is an increasing awareness of the potential for significant ethical risks in this regard with the use of AI, particularly concerning racial bias.^29^ For these practical and ethical reasons, an opt-out model is often preferred. As part of a review into the security and use of NHS data in the United Kingdom in 2016, the National Data Guardian recommended that a national opt-out model should be introduced, rather than one based on an opt-in consent.^30^ In 2020, clinical AI researchers from Stanford University proposed the use of a similar model for the development of AI in radiology.^19^

## Real-World Case Study

With ophthalmology at the forefront of AI-enabled health care and potentially acting as an exemplar for other medical specialties, the specialty has had to engage with these issues. For example, the collaboration between Moorfields Eye Hospital in the United Kingdom (led by P.A.K.) and DeepMind, an AI company, adopted a multipronged approach. First, it addressed an area with clear patient benefit, the development of a triage tool for macular diseases using optical coherence tomography (OCT) images.^31^ Second, all OCT scans used were de-identified to the standards described by the UK Information Commissioner's Office Anonymisation Code of Practice,^32^ as well as according to *BMJ* guidance for the sharing of clinical data.^33^ Third, contractual safeguards were put in place for non-exclusive data sharing that prohibits linkage with other datasets or attempts at re-identification. Finally, and most importantly, an active program of patient and public engagement was undertaken, with the aim of ensuring public transparency. This included early communication with the major eye disease charities and the Royal College of Ophthalmologists, as well as with the NHS Health Research Authority,^34^ as well as also providing information for those patients who preferred to opt out of the research, either at a local or national level.^30^

## Additional Legal Considerations

Many of the privacy concerns associated with Big Data and AI exist due to gaps in existing laws and regulations regarding traditional medical data. The Health Insurance Portability and Accountability Act (HIPAA) was enacted in 1996, prior to the rise of Big Data and AI. HIPAA regulates the use of protected health information (PHI) in the United States and requires de-identification of data by two mechanisms: (1) expert determination (expert risk assessment for a particular use) and (2) safe harbor (the removal of 18 prespecified identifiers).^35^ Whereas HIPAA is meant to ensure data protection on the part of healthcare providers and healthcare systems, these regulations may be inadequate for managing the ever-larger amounts of data associated with medical care today. For example, PHI can be shared without consent for treatment, payment, and operational purposes,^36^ and HIPAA does not cover data generated outside of health entities, such as patients, providers, and insurers, covered by the act.^2^ Examples of unregulated data include data from smart watches, mobile health applications, internet search engines, social media, and consumer-initiated health tests, such as genetic testing, all of which can be triangulated to re-identify individuals.^2^ In Europe, patients are protected under the General Data Protection Regulation (GDPR), which was implemented by the European Union in 2018 to regulate personal data protection. Under the GDPR, all health data are considered personal data, but there are exceptions under which health data may be used without consent such as for research purposes if safeguards are instituted.^37^ The GDPR introduced a weaker version of de-identification known as pseudonymization, which is the removal of only directly identifying data.^38^

## New Approaches

Some of the most promising approaches for protecting data privacy in the era of Big Data and AI are those that take advantage of the technology itself. One strategy, differential privacy, involves describing patterns of groups in the dataset rather than individuals.^39^ Federated learning (or collaborative learning) and distributed models are machine learning techniques that can be used to protect data by training algorithms across multiple servers using separate data samples.^40^^–^^42^ In this method, training code and models are brought into each data silo and trained in situ while the data remain in place. The combined model parameters, trained across many locations, would effectively have been trained on all available data without risking data breaches from allowing outsider use.

Similarly, training local generative adversarial networks (GANs)^43^ and then sharing GANs instead of data may mitigate re-identification risks.^44^^,^^45^ Each deep learning model would be trained to recapitulate the statistical distribution of the training set and would generate synthetic image examples that are different from the original images. This method would require each hospital to train an AI model to synthetically generate examples, and the resulting models would be transferred outside of the protected environments to generate synthetic images while still capturing disease-relevant imaging features. However, GANs have not been fully explored and must be applied with caution. An important metric for this approach is to ensure that the generated images are sufficiently different from the original images to preserve privacy. For example, the de-identification of a color fundus photograph may require that the resulting synthetic image cannot be re-identified by retinal vessel configuration^46^^,^^47^ or that the membership to the training set cannot be established. An important tradeoff is performance difference in the real world when models are trained with the original data compared with generated data that preserve privacy.^45^ If the generated synthetic datasets are too different from the original images, then the performance of the models trained with synthetic data would suffer and risk safety and efficacy when deployed.

Even when the data are not shared, there are other AI-related privacy issues that must be examined. Although individual patient data and imaging are safeguarded, traditionally the trained parameters or weights of AI models are not considered private or at risk for privacy breaches. However, large-parameter deep learning models, when trained with relatively few examples, can overfit and “memorize” these examples. AI models have been created to perform model inversion, where one AI model will attempt to reconstruct images with which another AI model was trained, which could potentially expose private data.^48^ Recently, even federated learning schemes with differential privacy have been overcome using GANs.^49^^,^^50^ Clearly, the tools that are developed to protect data privacy will have to adapt quickly as AI technology evolves. It is also clear that such tools cannot be utilized in isolation; careful consideration of ethical and legal frameworks, with the adoption of appropriate safeguards, will also be necessary. Furthermore, it is important to also consider potential unintended ethical consequences; for example, the use of GANs and federated learning could lock in incumbents who have the resources to develop such systems, thus inhibiting wider dissemination of clinical data for patient benefit.

## Conclusions

AI and Big Data have introduced privacy concerns that require solutions and updated regulations. New regulations governing privacy must be created to protect against inappropriate use of data, accidental disclosures, and weaknesses in de-identification techniques.^4^ However, we must also acknowledge that the overprotection of data may be detrimental to the data-driven innovation that ultimately improves our overall healthcare system.^2^ Ophthalmology has been at the forefront of AI development, but there is also much to learn from other medical specialties that have adopted AI and are also confronting these issues. A successful balance is possible as thoughtful solutions that can adapt to evolving technology are implemented. Education of our patients and the public, alongside transparency about usage and sharing, will become vital as this field rapidly matures.
